# AMMI and GGE Biplot Analyses for Mega-Environment Identification and Selection of Some High-Yielding Oat (*Avena sativa* L.) Genotypes for Multiple Environments

**DOI:** 10.3390/plants12173064

**Published:** 2023-08-25

**Authors:** Kibreab Yosefe Wodebo, Taye Tolemariam, Solomon Demeke, Weyessa Garedew, Tessema Tesfaye, Muluken Zeleke, Deribe Gemiyu, Worku Bedeke, Jane Wamatu, Mamta Sharma

**Affiliations:** 1 College of Agriculture and Veterinary Medicine, Jimma University, Jimma P.O. Box 307, Ethiopia; taye.tolemariam@ju.edu.et (T.T.); solomondemeke2000@gmail.com (S.D.); woyessa.garedew@ju.edu.et (W.G.); 2 Bonga Agricultural Research Center, Bonga P.O. Box 101, Ethiopia; 3 Arba Minch Agricultural Research Center, Arba Minch P.O. Box 2228, Ethiopia; tessema4@gmail.com; 4 International Centre for Agricultural Research in Dry Areas, Addis Ababa P.O. Box 5689, Ethiopia; m.zeleke@cgiar.org (M.Z.); j.wamatu@cgiar.org (J.W.); 5 South Agricultural Research Institute (SARI), Hawassa P.O. Box 06, Ethiopia; deribeg2000@yahoo.com (D.G.); bworku2002@gmail.com (W.B.); 6 International Crops Research Institute for the Semi-Arid Tropics, Hyderabad P.O. Box 502324, India; mamta.sharma@icrisat.org

**Keywords:** oat (*Avena sativa* L.), biomass yield, GXE Interaction, AMMI, GGE

## Abstract

This paper reports an evaluation of eleven oat genotypes in four environments for two consecutive years to identify high-biomass-yielding, stable, and broadly adapted genotypes in selected parts of Ethiopia. Genotypes were planted and evaluated with a randomized complete block design, which was repeated three times. The additive main effect and multiplicative interaction analysis of variances revealed that the environment, genotype, and genotype–environment interaction had a significant (*p* ≤ 0.001) influence on the biomass yield in the dry matter base (t ha^−1^). The interaction of the first and second principal component analysis accounted for 73.43% and 14.97% of the genotype according to the environment interaction sum of squares, respectively. G6 and G5 were the most stable and widely adapted genotypes and were selected as superior genotypes. The genotype-by-environment interaction showed a 49.46% contribution to the total treatment of sum-of-squares variation, while genotype and environment effects explained 34.94% and 15.60%, respectively. The highest mean yield was obtained from G6 (12.52 kg/ha), and the lowest mean yield was obtained from G7 (8.65 kg/ha). According to the additive main effect and multiplicative interaction biplot, G6 and G5 were high-yielding genotypes, whereas G7 was a low-yielding genotype. Furthermore, according to the genotype and genotype–environment interaction biplot, G6 was the winning genotype in all environments. However, G7 was a low-yielding genotype in all environments. Finally, G6 was an ideal genotype with a higher mean yield and relatively good stability. However, G7 was a poor-yielding and unstable genotype. The genotype, environment, and genotype x environment interaction had extremely important effects on the biomass yield of oats. The findings of the graphic stability methods (additive main effect and multiplicative interaction and the genotype and genotype–environment interaction) for identifying high-yielding and stable oat genotypes were very similar.

## 1. Introduction

The livestock subsector in Ethiopia significantly contributes to the national income [1] and rural and urban residents’ means of subsistence. However, because of a lack of feed and an imbalanced supply of feed, animal output remains poor [2]. The mean oat biomass production of 9.67 t/ha in this study is promising for developing nations like Ethiopia. This country is known for its lengthy dry season, during which there is inadequate plant biomass left over from the wet season to maintain domestic livestock species and key issues with feeding livestock arise [3].

*Avena sativa*, sometimes known as oat, is a significant multi-use cereal crop that is grown on more than 9 million hectares worldwide for grain, feed, fodder, and straw [4]. It is not very selective in terms of soils and climate and thus can reliably be grown in infertile soils and cool and humid climates [5]. One of the well-adapted and significant fodder crops that grow in Ethiopia’s highlands, primarily under rain-fed conditions, is *Avena sativa*. It is also one of the crucial fodder crops that are frequently cultivated in the winter, when animals confront a shortage of green fodder and the majority of the feed starts to deteriorate and eventually dry out [6]. In terms of cereal output, it comes in sixth place behind wheat, maize, rice, barley, and sorghum. Oats are excellent for making hay in climates that are suitable for them. Oats ensile effectively for use on farms. Compared with wheat or barley straw, oat straw is a more appealing and nutritious feed option for livestock. However, because there are not many breeding programs for fodder oats, cultivars are often developed and produced primarily for grain, and the same cultivars are used for both [7].

For quantitative variables like yield, a strong genotype-by-environment interaction might limit the relevance of inferences that would otherwise be true and significantly limit the ability to select superior genotypes [8,9,10]. The difference in the genetic ranking of genotypes in relation to the environment—for example, a genotype performing well in well-watered conditions but poorly in dry situations—is how [11] define genotype–environment interaction. The development of genotypes that can be adapted to a wide range of various settings is the ultimate goal of plant breeders in crop improvement efforts [11]. Finding genotypes whose performance remains stable in a variety of conditions can be accomplished using yield stability analysis [12,13]. The genotypes of oats that perform the best in target conditions and those that are most adaptable to other habitats can thus be found through comparing performance across environments.

Different statistical approaches for analyzing genotype stability can help with the difficult task of discovering superior genotypes in the context of significant G × E interactions [12].

The genotype, environmental factors, and their interaction have a considerable impact on yield and characteristics [14,15,16]. According to [15], genotype–environment interactions (GEIs) cause genotypes to behave differently in various settings. Breeders want to determine the best growing conditions for their genotype in addition to quantifying the GEI [16]. An ideal stable genotype is one that consistently produces good results for agronomic and quality factors over a wide range of environmental conditions. According to [17], the genotype–environment interaction is usually assessed according to the AMMI (additive main effects and multiplicative interaction) model and GGE biplot analyses in order to predict phenotypic responses to environmental changes in the examined genotypes. In the AMMI approach, principal component analysis (PCA) with multiplicative parameters and analysis of variance (ANOVA) with additive parameters are combined into a single analysis. The main and interaction effects for environments and genotypes are displayed together in the AMMI biplot. Additionally, Ref. [18] offered a solitary examination of the genotype-by-environment interaction.

Powerful methods for examining and providing commentary on multi-environment data structures in breeding operations include the AMMI and GGE biplot models [19,20]. Researchers interested in agriculture are particularly interested in these two statistical analyses (AMMI and GGE). This is due to the fact that they may be applied to any two-way data matrix, even those involving a number of genotypes examined across many sites [21]. These analyses include principal component analysis (PCA) and analysis of variance (ANOVA) [22]. The GGE biplot analysis and the AMMI biplot analysis differ in that the former is based on an environment-centered PCA, while the latter is based on a double-centered PCA [23].

The majority of genotypes have been reported to exhibit narrow adaptability and significant genotype-by-environment interaction (GEI) effects, despite the fact that oats typically adapt to a broad range of environmental scenarios [24,25]. The significance of extending research efforts to examine the variations in biomass yield across oat genotypes and across settings was studied in this report. This study will help researchers plan the further breeding and production of promising, specific, and widely adapted genotypes. The examination of the performance of oat genotypes in various conditions in Ethiopia is still in its infancy. Therefore, the objectives of this study were to (1) estimate the magnitude of the genotype-by-environment interaction, (2) identify stable genotypes with a high biomass yield based on dry matter, and (3) identify mega-environments to guide future testing strategies.

## 2. Results and Discussion

### 2.1. AMMI ANOVA

Eleven oat genotypes were examined in four locations using the AMMI model, and the results showed that the environment (E), genotype (G), and genotype–environment interaction (GEI) all had a significant (*p* < 0.001) impact on the yield of oat (t ha^−1^).

AMMI (IPCA1) was highly significant (*p* < 0.001) according to the AMMI model’s analysis of variance (Table 1). This showed that strong genotype-by-environment (GE) interaction caused a significant difference in yield performance among the oat genotypes across the studied environments. As a result, it might be possible to create stable genotypes or entries for a certain environment. This discovery is consistent with several studies that have discovered significant interactions between the environment and genotypes of oats [24,25,26].

Oat biomass dry matter yield was influenced by the genotype-by-environment (GE) interaction effect (49.46%), genotype effect (34.94%), and environment effect (15.60%), according to the total percentage explained by sum-of-squares factors (Table 1). The genotypic response differed significantly among environments, as seen by the fact that the GEI sum-of-squares component was greater than the genotype sum-of-squares factor. Since the GE interaction or the sum of squares contributes more to the overall variance, there is a greater likelihood that cultivars will evolve for a particular environment. In line with these findings, Ref. [25] revealed that the genotype-by-environment interaction effect, followed by genotype and environment, contributed to the biggest overall sum of squares. The result is in contrast to Adjebeng-Danquah [27], who reported that the environment contributed a greater proportion of the treatment sum of squares, followed by the genotype and genotype-by-environment interaction. This was in contrast to findings from [26], who reported that the environment is the most contributing, followed by the genotype-by-environment interaction effect and the genotype effect. Both studies found that environmental conditions had a significant impact on production. This study result shows the yield greatly depends on the environment. However, genotype (G) and genotype-by-environment interaction (GEI) are relevant to genotype evaluation, whereas the significant environmental influence is irrelevant [26].

According to [18], the first two IPCAs can be used to forecast the AMMI model that is the most accurate. Several authors took the first two IPCAs for GGE biplot analysis because the greater percentage of genotype-by-environment interaction (GEI), in most cases, were explained by the first IPCA, such as for maize [28], bread wheat [29,30], common bean [29], for finger millet [30], field pea [31] and oat [14,32].

The AMMI with IPCA1 and IPCA2 is the most effective predictive model for the cross-validation of the yield variation explained by the GEI [33]. Considering IPCA1 (73.43%) and IPCA2 (14.97%) together, the sums of squares from IPCA1 and IPCA2 contributed 87.43% of the total GEI, with the IPCA1 having a larger sum of squares than genotypes. The entire genotype-by-environment interaction component was adequately explained by the model [34]. Given that it removes the bulk of the real variation, this suggests that the AMMI model with the IPCA1 and IPCA2 were suitable for cross validating the oat biomass dry matter yield variation supplied by GEI in the given data set. The GEI contributes more than genotypes, suggesting a need for research into the basis of the diverse ways that genotypes respond to their surroundings (Table 1). Because GEI weakens the usefulness of genotypes through mystifying their yield performance by means of decreasing the relationship between genotypic and phenotypic characteristics [35,36], GEI complicates the selection process. 

The average genotype biomass dry matter yield ranged from 8.65 t ha^−1^ (ILRI_15152A = #G7) to 12.52 t ha^−1^ (ILRI_5527A = #G6), while the average environment biomass dry matter yield ranged from 8.50 t ha^−1^ at Hulla to 10.67 t ha^−1^ at Adiyo (Table 2). As shown by genotype yield rankings that varied between environments, with the exception of genotypes G6 and G5, some GEI genotypes were of a crossover type (Table 2). The highest biomass dry matter yield across conditions was consistent for genotypes G6 and G5. Thus, the top-ranking genotypes in Chencha, Adiyo, Doyegena, and Hula, respectively, were genotypes G6 and G5 (Table 2).

### 2.2. AMMI Biplot

**Figure 1:**
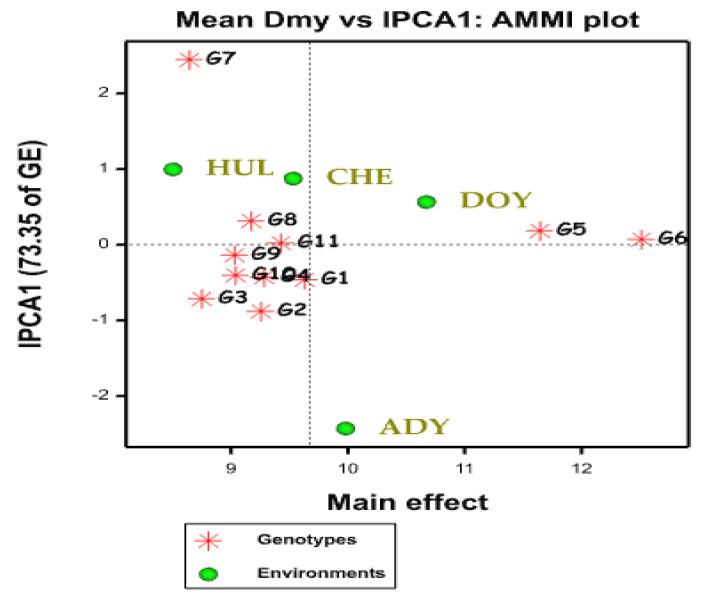
Graphics of AMMI biplot of dry matter yield of oat genotypes using symmetrical scaling of both genotypes and environments. HUL, CHE, DOY, and ADY stand for Hulla, Chencha, Doyogena, and Adiyo, respectively.

Four parts make up the AMMI1 biplot space (Figure 1), ranging from high-yielding environments in parts 2 (upper right) and 3 (low right) to low-yielding environments in sections 1 (higher left) and 4 (low left). The biplot in Figure 1 clearly shows that the points for the genotypes are more dispersed than the points for the environment, indicating that genotype variability is greater than environment-related variability, which is consistent with ANOVA (Table 2). The points for the typically adapted genotypes on the biplot would be close to the IPCA = 0 line (which shows negligible or no GE interaction) and on the right side of the grand mean levels (suggesting high mean performance). In this regard, the AMMI biplot was set up with two oat genotypes, such as G6 and G5, with two environments, such as Doyogena and Adiyo, on the right side of the perpendicular vertical line (Figure 1).

The oat genotypes (G5, G6, G9, and G11) are weakly influenced by environmental factors (lower interaction effect). The genotypes’ (G2, G3, and G7) dry matter yield was strongly influenced by environmental factors (higher interaction effect), as shown in Figure 1. However, the dry matter yield response may not be higher for the genotypes that were less sensitive to environmental influences.

Oat genotype ILRI_5527A (G6), which was closest to the IPCA 0, was more adaptable, high-yielding, and stable throughout the tested settings, so it was excellent. Along with G6, G5 also achieve a high dry matter yield and was adaptive in all of the studied situations. In contrast to the optimum genotype ILRI_5527A9G6), the genotypes G7, G2, and G3 were far from the IPCA 0 of the biplot and yielded low dry matter (Figure 1).

Genotypes close to the IPCA 0 of the biplot are not susceptible to environmental interaction, whereas genotypes further from the origin of the biplot are sensitive and have significant interaction effects, according to [19,35,37]. Additionally, Ref. [35] claims that the best genotypes have small absolute IPC2 scores (great stability) and large IPC1 scores (wider adaptability). Figure 1 shows how the environments Adiyo and Hulla differed from Doyogena and Chencha in terms of the genotypes’ dry matter yield performance. According to [36], environments with short spokes impose weak interacting pressures on the performance of oat genotypes, whereas settings with long spokes exert high interaction.

On the other hand, certain environment (Figure 1) stood out as having a modest, moderate, or significant contribution to the interaction (Doyogena), Chencha, Hulla, and Adiyo, respectively. These environments (Adiyo and Doyogena) produced a mean dry matter yield that was higher than the overall mean (9.67 t ha^−1^), demonstrating that they were the best places to find high means. The settings with the highest potential (Hulla) showed variable genotype performance for dry matter yield and had a strong positive IPCA1 score (Figure 1). All genotypes progressed poorly in the low-yielding environment (IV), which had the lowest yield but a negative IPCA1 score. Similar observations were reported by different authors [36,38]. Settings with varying contribution relationships in both high-yielding and low-yielding conditions were also part of different observed findings [39].

### 2.3. AMMI Stability Value (ASV)

An AMMI stability value was computed to determine the stability of the genotypes (Table 3). ASV is the distance from zero in a two-dimensional scatter graph comparing IPCA1 (interaction principal component analysis axis 1) scores to IPCA2 scores. The difference in stability measurements between the two primary components can be made up for using the proportional difference between the IPCAs (1:2), which can then be calculated using the Pythagorean theorem to account for the AMMI stability value [40]. The AMMI stability value (ASV) quantifies and ranks genotypes based on their yield stability rather than providing a quantitative stability indicator [38].

In this respect, greater ASV values are associated with unstable genotypes, whereas genotypes with lower ASV values are associated with more stable genotypes. The genotypes G7, G2, and G3 were the most unstable, with ASV values of 4.9, 1.79, and 1.45, respectively (Table 3). Genotype G9 was the most stable, with an ASV value of 0.27, followed by genotypes G6 and G11, with ASV values of 0.29 and 1.08, respectively, in biomass dry matter. Refs. [13,41,42,43] all employed a similar technique and discovered a more stable genotype with a lower ASV value.

### 2.4. Genotype Selection Index (GSI) Analysis

There is a need for methods that combine mean and stability into a single criterion since the most stable genotypes may not always produce the best yield performance. Stability should not, however, be the primary parameter for selection. In this regard, because ASV considers both IPCA1 and IPCA2, which account for the majority of the variation in GE interaction, the rank of ASV and rank of mean yield are combined to form the Genotype Selection Index (GSI), a single selection index [41]. Because the most stable genotypes may not always produce the highest yields, stability is not the main factor in selection. Only when it is connected to average performance does the phrase “high stability” have any real meaning [42]. Therefore, methods that combine mean yield and stability into a single indicator are required [43]. The lowest GSI value, with a high mean yield, is regarded as the most stable.

The smallest GSI is considered the most stable (Table 3); in that regard, the most stable genotypes with the highest biomass dry matter yield were G6 and G5, which had the lowest GSI values of 3 and 6, respectively. These genotypes were followed by G11 and G4, which had GSI values of 7 and 12, respectively, suggesting that they were stable and high yielding. This result is consistent with other research that found stable genotypes with high yields could be found through analyzing the genotype selection index based on ranking mean yield and ranking AMMI stability value [41,42,43]. These outcomes matched those of the biplot graph.

### 2.5. GGE Biplot

#### 2.5.1. Which-Won-Where View of GGE Biplot

**Figure 2:**
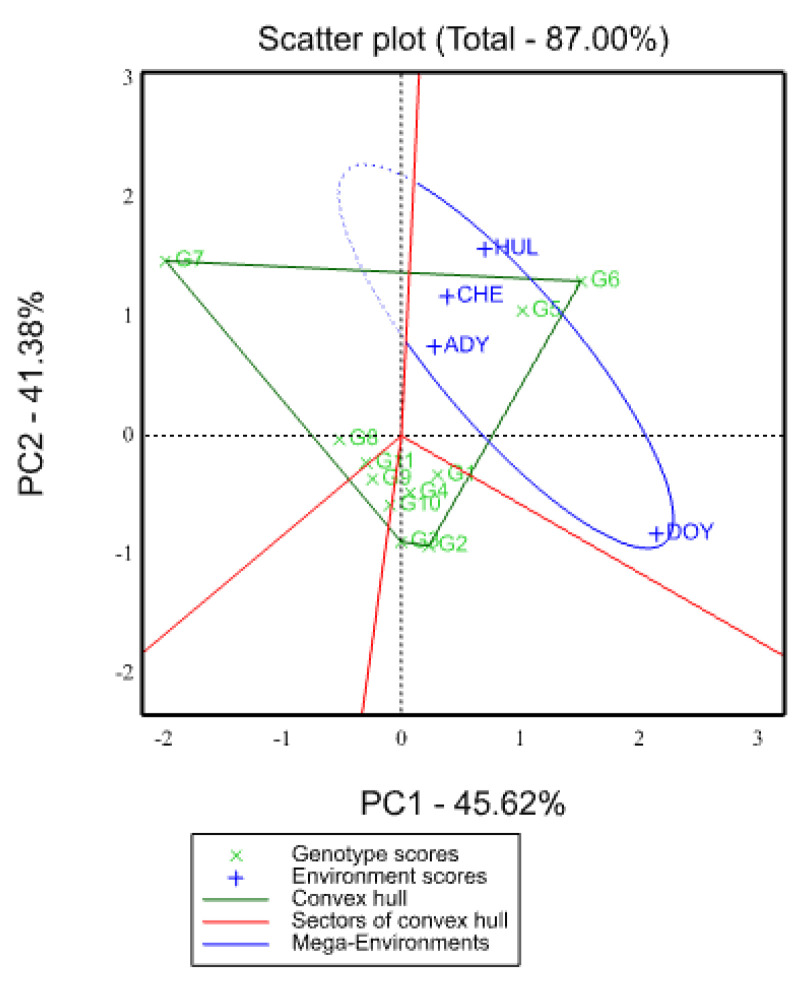
The GGE biplot shows which genotype won where and their related mega-environments. HUL, CHE, DOY, and ADY stand for Hulla, Chencha, Doyogena, and Adiyo, respectively.

In order to identify the winning genotypes through showing the patterns of interaction between genotypes and environments, a polygon view of the GGE biplot graphic analysis is shown (Figure 2) [34]. In multi-location yield experiments, it is useful for detecting crossover and non-crossover genotype-by-environment interactions as well as the potential existence of several mega-environments [14,20]. G2, G3, G6, and G7 were the vertex genotypes, as shown by the genotypes in Figure 2. Because they are the farthest from the origin of the biplot, these genotypes perform better or worse in some or all environments [34], and they are regarded as specifically suited genotypes because they are more responsive to environmental change. In the GGE’s polygon-view biplot, they thrive in environments that are a part of their particular sector [34].

The two genotypes that performed best in each of the four environments—Hulla Chencha, Adiyo, and Doyogena—were G6 and G5. On the other hand, because they were located on the other side of the test environments and the furthest from the biplot’s origin, the vertex genotypes—except G6, the rest: G2, G3, and G7—were the ones that performed the poorest over practically the entire set of test settings. Similar findings reported on genotypes’ which-won-where patterns [26,44,45,46] agree with this study’s finding. They discovered that certain genotypes performed better than others in a particular environment and that certain genotypes performed worse in certain contexts. The red-color equality lines in Figure 2 separate the biplot into four portions. While the genotypes were spread over all four sectorial areas, the environments were only scattered across one. These were only one mega-environment: Hulla, Chencha, Adiyo, and Doyogena. This implies that similar genotypes perform well in an environment of homogeneity. In order to manage the genotype-by-environment interactions and subsequently relate the findings to similar agro-climatic regions, the identified mega-environments may be helpful. The most productive genotypes in a sector are those that are located close to the vertex [16]. The first (I) sectors, which were devoid of any environment, included seven and eight genotypes, respectively (Figure 2). In the second sector (II), there were four environments that were found: Hulla, Chena, Adiyo, and Doyogena. The G5 and G6 genotypes were included in this region (Figure 2). G6 is the vertex (winner) at the four environments of the sector. The third sector (III) contained four genotypes (G1, G2, G3, and G10) without any environments; G2 and G3 were the vertex genotypes, indicating that these were the better genotypes for sector III (Figure 2). Without any environments, G9 and G11 were located in the fourth sector (IV), with no vertex genotype in this sector. The GEI variance was higher in the vertex genotypes than in the genotypes close to the origin. Resulting around average performance, the G1, G4, G5, G8, G9, G10, and G11 genotypes were close to the biplot origin, and their GEI variation was lower than that of the vertex genotypes. The results were similar to those reported by [40,42,47,48,49,50], who stated that the testing environment was divided into various mega-environments with winning genotypes and sectors with different numbers of genotypes. The effects of GE interaction influence the accuracy of predicting the performance of some genotypes in new environments. This was observed for genotypes ranked third and above, for example, genotype eleven (G11), which is ranked as third in the environment of Chencha but not found within the top-five ranking in the Doyegena environment. Similarly, genotype two (G2) ranked third in Doyegena but was not found among the top five in Chencha and Hulla. The results show new promising genotypes (G6 and G5) which were high yielding and stable in all environments, which is recommended for the breeder for further production.

#### 2.5.2. Relationship among Environments

**Figure 3:**
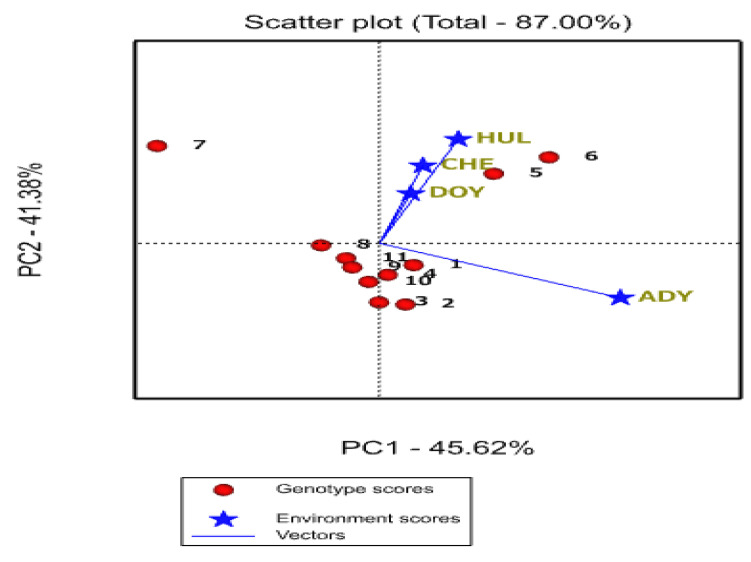
The GGE biplot graph shows relationships among test environments, where 1 up to 11 stands for genotypes from 1 up to 11, consecutively. HUL, CHE, DOY, and ADY stand for Hulla, Chencha, Doyogena, and Adiyo, respectively.

According to Figure 3 of the GGE biplot, the first (PC1) and second (PC2) principal components together accounted for 87% of the total variation, showing that this biplot may be utilized to distinguish between interrelationships across the environments. The correlation coefficient is related to the angle between the biplot origin and the test environment markers [35]. Additionally, a high degree of genotype discrimination is conferred by the length of an environmental vector [19]. In the current study, Doyogena was the environment that was most discriminating (held more information) about the genotypes with the longest vectors from the origin, followed by Hulla and Chencha, which were moderately discriminating, and the Adiyo environment, which was either barely or not discriminating about genotype differences (Figure 3).

The use of non-discriminating (non-informative) test environments is discouraged since they offer little information regarding genotypes [42]. Additionally, test environments with acute, obtuse, and right-angle relationships, respectively, have positive, negative, and zero correlation between environments predominantly found using the biplot vector view [16].

The four environments were divided into two groups based on the angle test between environment vectors. Figure 3 shows that the first group had a modest angle between the environments Hulla, Chencha, and Adiyo; a strong positive correlation between them; and similar genetic information. It means that their capacity to distinguish between genotypes is compromised by the environment. Breeders should be able to use fewer test environments, lowering testing costs and increasing breeding effectiveness, if they can obtain trustworthy information about environment similarity and clustering. The second group is in possession of the broad angle between Doyogena and the rest of the environments.

The performance of the genotype in an environment is better than average if the angle between its vector and the environment’s vector is less than 90. And, it is less than average if the angle is greater than 90° and near average if the angle is about 90° [16]. In this regard, G6 and G5 performed well in environments Chencha, Hulla, Adiyo, and Doyogena. Wide obtuse angles are a sign of strong crossover GE in the test conditions, and the biggest angle is somewhat wider than 90°, suggesting that the GE is moderately large. Therefore, except for G6 and G5, the rest of the genotypes showed varying performance in different environments. Similar findings in the link between environments defined using the angle method were reported by [40,51,52]. When compared to associations with small angles, which showed significant positive correlations and provided information about genotypes, they discovered that some settings between them had huge angles or low or negative correlations.

#### 2.5.3. Evaluation of Genotypes Based on the Ideal Genotype

**Figure 4:**
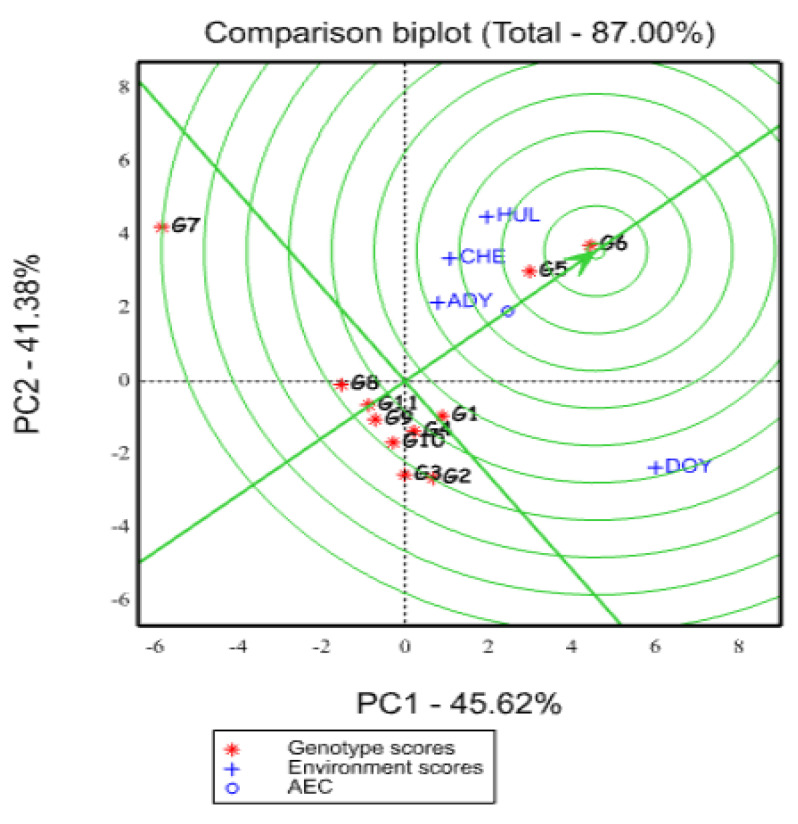
GGE biplot graph based on genotypic focused scaling for comparison genotype with ideal genotype represented by the center of concentric circles and an arrow pointing to it. HUL, CHE, DOY, and ADY stand for Hulla, Chencha, Doyogena, and Adiyo, respectively.

An interesting application for comparing genotypes to a desirable genotype is the GGE biplot model. An ideal genotype has a high mean performance as well as a high level of stability across locations; a number of publications, including [18,45,53,54], claim that the optimal position—the center of the concentric circle—was used to identify the genotype with the highest mean performance and stability. Large PC1 scores (high mean yield) and small (absolute) PC2 scores (high stability) characterize an optimal genotype. Even though such an “ideal” genotype could not exist in the real world, it might be utilized as a standard for assessing genotypes [52]. To more clearly show the disparity between genotypes and the ideal genotype, concentric circles were created in a GGE biplot graph based on genotype-focused scaling [18,23]. Early breeding cycles can be used to rule out genotypes that are quite different from the ideal genotype, whereas later testing can take into account genotypes that are relatively similar to it [55]. When a genotype is nearer to the “ideal” genotype, which is shown in the first concentric circle of the GGE biplot graphic, it is considered to be more desirable [56]. The genotype G6 was located in the first concentric circle, as shown in the GGE biplot graph (Figure 4). It follows that G6 was the preferred genotype position, followed by G5, making it the preferred genotype among those of oat.

This is comparable to the findings of [35,44,54] who showed that the first and second concentric circles, respectively, include one optimal genotype and a few other suitable genotypes. Similar to this, Ref. [26] found desired genotypes using various strategies. Their criteria were that an ideal genotype should have big PC1 scores (high mean yield) and a small absolute PC2 score (high stability); however, their strategy was unsuccessful in doing so. The highest-yielding genotypes are those that are drawn on the concentric and/or average environmental coordinate (AEC) center closest to the ideal genotype [57,58].

In the consideration of AMMI and GGE biplot analysis of oat genotype based on the dry matter yield performance, genotypes G6 (ideal genotype) and G5 yielded more dry-matter biomass than the remaining tested oat genotypes across the tested environments. Therefore, these genotypes are relatively wider in adaptation across the tested environments.

#### 2.5.4. Evaluation of Environments Relative to Ideal Environments

**Figure 5:**
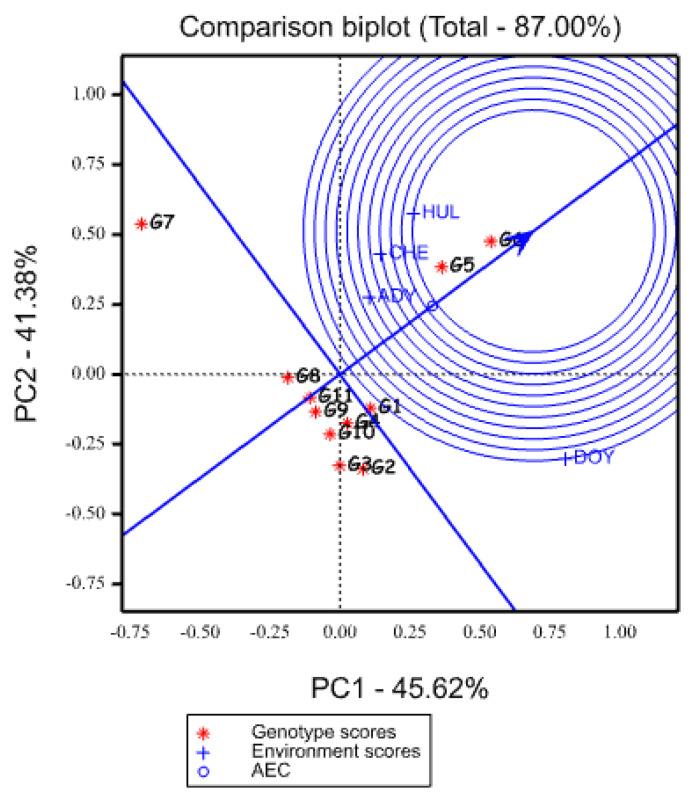
GGE biplot graph based on environment focused scaling for comparison of an environment with an ideal environment represented by the center of concentric circles and an arrow pointing to it. HUL, CHE, DOY, and ADY stand for Hulla, Chencha, Doyogena, and Adiyo, respectively.

The desired environments are those that are closest to the ideal environment, which is located in the first concentric circle of the environment-focused GGE biplot. Hulla has been in the perfect environment and is in the first concentric circle in this regard (Figure 5). Hulla had a robust PC1 score but a reduced PC2 score. Therefore, genotype evaluation in the Hulla environment increased the observed genotypic diversity across genotypes for the biomass dry matter yield of the tested oat genotypes and should be regarded as the most appropriate to identify broadly suited genotypes. Chencha’s and Adiyo’s habitats have been recognized as desirable environments (Figure 5), since they are quite similar to the ideal environment (Hulla).

The Doyogena environments, on the other hand, were placed far from ideal conditions, making them unsuitable (fewer representatives) environments for choosing cultivars with broad adaptations but useful for choosing those with particular adaptations. Soil fertility, rainfall, and other environmental variability between environmental systems can be linked to this variation. The discriminating power of a place is influenced by the genotype composition, but the existence of GEI makes selecting a suitable test location more challenging [35]. Furthermore, they suggested that the most representative environments can be used for widely adapted genotype selection, while non-representing environments can be useful for specifically adapted genotype selection.

#### 2.5.5. Ranking of Genotypes Based on Mean Yield and Stability Performance

**Figure 6:**
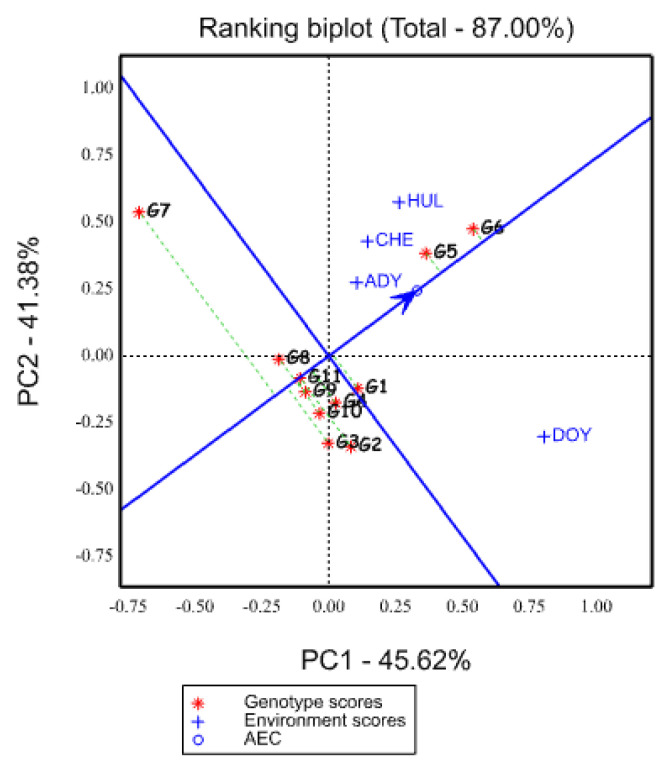
Average environment coordinates view of the GGE biplot graph showing the ranking of genotypes represented by an arrow pointing to it for mean biomass yield based on dry matter and stability performance over environments. HUL, CHE, DOY, and ADY stand for Hulla, Chencha, Doyogena, and Adiyo, respectively.

The average environment (tester) coordinate (AEC) methods were used in the GGE biplot to determine mean dry matter yield and genotype stability (Figure 6) [59]. The average PC1 and PC2 scores across all environments are used to define the average environmental (tester) coordinate (AEC) [34]. The mean dry matter yield performance axis of genotypes is indicated by the AEC X axis (PC1) line that goes through the biplot’s origin and has an arrow pointing in the direction of the positive end. The stability of genotypes (PC2) is measured using the line which runs through the origin and is perpendicular to the average environmental axis (Figure 6). Stable genotypes had PC2 scores that were practically zero, were close to AEC (PC1), and had the lowest number of perpendicular lines. Away from the biplot origin, however, any direction on the axis denotes a greater GE interaction and a lower level of stability.

The best genotypes for selection criteria are those with both high mean yield and high stability. In this regard, in the present study (Figure 6), the single arrowed line pointed to higher yield across environments. Therefore, genotype G6 had the highest mean yield, followed by G5. They were the most stable, while G7, G3, and G2 were highly unstable, and among them, G7 performed poorly. The present study’s findings are in line with the report made by [60]. They ranked genotypes based on mean performance and stability across environments. In this way, they found some genotypes to be the most stable with a high mean yield and some unstable high yielders, while some other genotypes were unstable with a poor yield and a stable low yielder.

The AMMI model has been successfully applied in many studies to analyze GEI [32,35]. The AMMI model, put out by [61], makes use of analysis of variance and principal component analysis for a better understanding of GEI, its causes, and effects. The GGE biplot analysis, which prioritizes both genotype main effects and GEI effects for the study, was proposed by [59]. Only the beginning analysis steps—where GGE examines G plus GE (or GEI) while AMMI separates G from GE—and the final analysis processes, where the biplot for the interpretation are built, distinguish these models from one another. The environmental stratification provided by the AMMI biplot is complemented by the GGE biplot, which enables the identification of mega-settings and genotypes that perform best in these environments [62]. These distinctions, however, do not suggest that either approach is better than the other [58]. The graphic analysis offered by the AMMI biplot offers a very straightforward analysis for breeding researchers. Conclusions about phenotypic stability, genotype behavior, genetic divergence between genotypes, and environments with the best performance can be made based on the data. Accordingly, the stable and high-yielding genotypes G6 and G5 are advised for breeding researchers to use in breeding programs.

## 3. Materials and Methods

### 3.1. Description of Study Area

**Figure 7:**
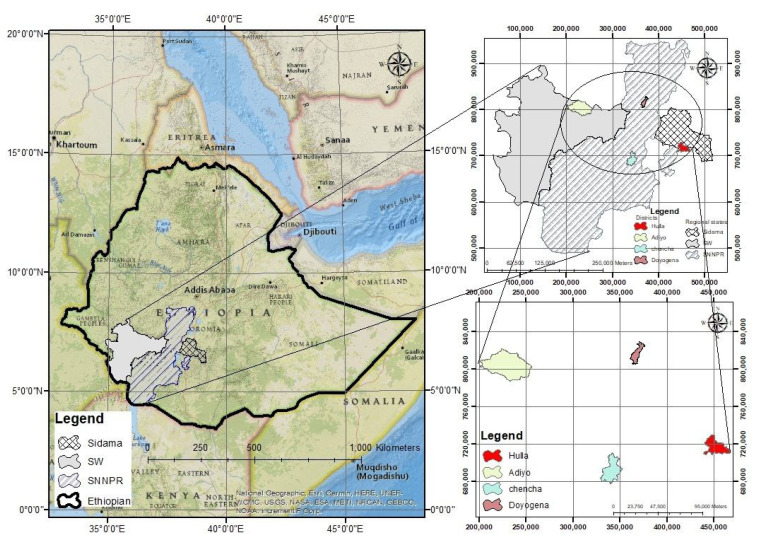
Map of the study area.

During the primary growing season of 2018–2019, the field experiment was carried out in four different environments. The study areas are shown in Figure 7.

The soil type, altitude, and mean annual rainfall at these locations are different from each other (Table 4). Consequently, each place was regarded as having its own ecosystem and considered an individual environment.

### 3.2. Experimental Materials

Eleven oat genotypes used in the study are displayed in Table 5. From the total genotypes, SRCPX80AB2291 and SRCPX80AB2806 were released, and the rest were promising.

### 3.3. Experimental Design and Management

The experiment was laid out in an RCBD design, with three replications. The design had three blocks and there were 33 plots, each with a dimension of 2 m × 3 m and a plot area of 6 m^2^. The space between rows, blocks, and plots was 20, 150, and 100 cm, respectively. Each plot had 10 rows. The experimental seeds were planted through drilling into the soil. Each plot requirement of the seeds was measured through calculating the oat seed plantation rate of 100 kg/ha. NPS and urea fertilizers were applied at 100 kg/ha each. Data on biomass yield at the milk stage were collected from the three central rows excluding the outside rows; it was weighed and then converted to tons per hectare (t ha^−1^).

### 3.4. Data Analysis

Utilizing Genstat, as reported by [63], statistical analyses were carried out. Each environment’s data were subjected to an analysis of variance (ANOVA) and a normality test prior to performing the combined analysis of variance across environments. After validating the homogeneity of the variances, the combined analysis of variances across sites was carried out. Bartlett’s tests of homogeneity of variances were used to determine the homogeneity of the error variances of the individual location experiments. With genotypes acting as fixed factors and surroundings acting as random variables, a combined ANOVA was created using the AMMI model.

#### 3.4.1. AMMI Analysis

The AMMI model’s biomass dry matter yield was examined. AMMI stands for additive main effect and multiplicative interaction. Because in the validity test, the MS component of the RCBD design for the block within replication is less than the residual error across all sites, the analysis of variance was a combined analysis based on the RCBD. According to [64], the AMMI analysis was utilized to modify both the multiplicative effects of the GE interaction by the principal component analysis and the main or additive genotype and environmental effects by analysis of variance. The following model was proposed by [64] for the AMMI analysis of variance (ANOVA):

(1) $$Y_{ij}=\mu +G_{i}+E_{i}+\sum_{k=1}^{n}\gamma_{k}\alpha_{ik}\gamma_{jk}+\varepsilon_{ij}$$

where *Y_ij_* is the mean yield of the *i*th genotype in the *j*th environment;

μ is the grand mean;

*G_i_* and *E_j_* are the genotype and environment deviation from grand mean, respectively;

$\alpha_{ik\;}and{\;\gamma }_{jk}$ are the genotype and environment principal component scores for axis *k*;

*n* is the maximum number of multiplicative terms;

$\gamma_{k}$ is the *k*th singular value of x (square root of the eigenvalue of xx’ or x’x);

$\varepsilon_{ij}$ is the error term.

#### 3.4.2. AMMI Stability Value (ASV) Analysis

Because the AMMI analysis does not offer a quantitative measure of stability, Purchase et al.’s [40] recommendation was to utilize an ASV measure to quantify and categorize genotypes according to their yield stability. The stability of a genotype is evaluated using the ASV. Weighted IPCA1 and IPCA2 scores indicate that the stronger the stability, the lower the value [40]. The following formula was used to determine the ASV.

The AMMI stability value (ASV) as described by [40] was calculated as follows:

(2) $$ASV=\sqrt{{\left[\frac{{IPCA1}_{sum\;of\;square}}{{IPCA2}_{sum\;of\;square}}\left({IPCA1}_{SCORE}\right)\right]}^{2}+{\left({IPCA1}_{score}\right)}^{2}}$$

where $\frac{{SS}_{IPCA1}}{{SS}_{IPCA2}}$ is the weight given to the IPCA1 value through dividing the IPCA1 sum of squares by the IPCA2 sum of squares. The larger the IPCA score, either negative or positive, the more specifically adapted a genotype is to certain environments. Smaller ASV scores indicate a more stable genotype across environments.

#### 3.4.3. Genotype Selection Index (GSI) Analysis

The genotype selection index was calculated using the equation GSI = RASV + RY [43]. The terms RASV and RY in this context stand for genotype mean yield ranking across environments and AMMI stability value ranking, respectively. The author claims that GSI combines stability and mean yield into a single criterion, with a low score suggesting stable genotypes with a high mean yield. Therefore, it is assumed that the GSI with the lowest value is the most stable and has the maximum biomass dry matter yield. A genotype is better suited to particular surroundings when it has a higher IPCA score, whether positive or negative.

#### 3.4.4. Gene Gene Environment Biplot

Based on the singular value decomposition of the first two principal components, the model for a GGE biplot [35] is

(3) Yij−μ−βj=λ1ξi1−ηj1+λ2ξi2−ηj2+εij

where $Y_{ij}$ is the mean for the *i*th genotype in the *j*th environment, µ is the grand mean, *βj* is the main effect of environment *j*, *λ*_1_ and *λ*_2_ are the singular values of the first and second principal components (PC1 and PC2), *ξi*1 and *ξi*2 are the PC1 and PC2 scores, respectively, for genotype *i*th, *η_j_*_1_ and *η_j_*_2_ are the eigenvectors for the *j*th environment for PC1 and PC2, and ε is the residual error term.

## 4. Conclusions

The findings of this study support the need to test genotypes in representative environmental settings in order to find the most stable and productive genotypes. To lessen the impact of GE interaction and to increase the precision and refinement of genotype selection, the yield and stability of performance should be taken into account simultaneously. The genotypes, environments, interaction of genotype × environments, and AMMI component were significant in the analysis of variance for the AMMI model of oat biomass yield based on dry matter. Therefore, it is important to include yield along with PCA1 and PCA2 scores at the same time in order to maximize the useful effects of GEI and increase the accuracy of genotype recommendations. It was possible to find genotypes with superior and consistent biomass yield based on dry matter production using a graphical interpretation of the AMMI analysis and GSI index, which combined the ASV and the yield potential of various genotypes into a single non-parametric index. Based on YSI or GSI indices, G6 and G5 revealed the highest yield and stability. Generally, AMMI analysis is advantageous for identifying high-yielding and stable genotypes (G6 and G5), whereas GGE is advantageous for identifying genotypes that are specifically or broadly adapted. According to GGE, the same genotypes (G6 and G5) were broadly adapted. The two methodologies of analysis (AMMI and GGE) approve selecting G6 and G5 for a further breeding program or for high-production growers in the study area. This study may need to be repeated after a number of years because there will be a change in environments over a number of years, which is a challenge of this study. A limitation of the study is that the graphical analysis of GGE estimates about 87% of the reason, not 100%; i.e., a greater proportion could be explained with a better alternative methodology.

## Figures and Tables

**Table 1:** Combined analysis of variance from AMMI model for dry matter yield of genotypes.

Source	D.F.	S.S.	M.S.	(%) SS Explained
Total	263	3052.9	11.61	
Treatments	43	1052.3	24.47 ***	
Genotypes	10	367.6	36.76 ***	34.94
Environments	3	164.2	54.72 **	15.60
Block	8	92.7	11.59	
Interactions	30	520.5	17.35 **	49.46
IPCA 1	12	381.8	31.81 ***	73.35
IPCA 2	10	77.9	7.79	14.97
Residuals	−6	0	0	
Error	212	1907.9	9	

Key: *** *p* ≤ 0.001 and ** *p* ≤ 0.05.

**Table 2:** Combined analysis of variance for biomass yield under different locations (t/ha).

Genotypes		Environments				Genotypic Mean	Rank
Code	Name	Chencha	Adiyo	Doyogena	Hulla		
G1	ILRI_5431A	8.30	10.26	11.11	8.85	9.63	3
G2	ILRI_5444A	7.08	11.38	11.55	7.02	9.26	6
G3	ILRI_5490A	9.47	7.88	10.93	6.72	8.75	10
G4	ILRI_5499A	8.89	9.75	10.67	7.82	9.29	5
G5	ILRI_5526A	11.51	11.67	11.65	11.75	11.65	2
G6	ILRI_5527A	12.48	12.65	12.77	12.17	12.52	1
G7	ILRI_15152A	10.47	11.11	3.01	9.99	8.65	11
G8	ILRI_15153A	8.99	11.38	8.60	7.72	9.17	7
G9	ILRI_16101A	8.75	9.88	9.69	7.82	9.03	9
G10	SRCPX80AB2291	8.23	10.09	10.31	7.53	9.04	8
G11	SRCPX80AB2806	10.69	11.36	9.52	11.36	9.43	4
Environmental Mean		9.53(3)	10.67(1)	9.98(2)	8.50(4)	9.67	
LSD (5%)		2.81	2.88	3.55	3.12		
CV (%)		15.46	14.93	18.75	21.72		
F value		**	***	*	**		

Key: *** *p* ≤ 0.001, ** *p* ≤ 0.05, and * *p* ≤ 0.1.

**Table 3:** Grand mean biomass yield based on dry matter (BDMY) tha^−1^, RY, GSI, ASV, RASV, IPCA1, and IPCA2 of 11 oat genotypes across environments.

Genotypes	BYDM	RY	GSI	ASV	RASV	IPCA1	IPCA2
G1	9.63	3	11	0.937532498	8	−0.46271	0.32331
G2	9.26	6	16	1.793726372	10	−0.88151	−0.73616
G3	8.75	10	19	1.45376979	9	−0.7156	0.56232
G4	9.29	5	12	0.86520405	7	−0.42998	0.16699
G5	11.65	2	6	0.492768176	4	0.18251	0.80585
G6	12.52	1	3	0.290958358	2	0.06914	0.62493
G7	8.65	11	22	4.900847781	11	2.4432	−0.01007
G8	9.17	7	12	0.679687821	5	0.314	−0.62413
G9	9.03	9	10	0.277265348	1	−0.13761	0.06356
G10	9.04	8	14	1.982438484	6	−0.40402	−0.09492
G11	9.43	4	7	1.08732656	3	0.02258	−1.08168

DMY: dry matter yield; RY: rank of yield; ASV: AMMI stability value; RASV: rank of AMMI stability value; GSI: genotype selection index.

**Table 4:** Description of test environments.

Location	Altitude (m.a.s.l)	Annual Av RF (mm)	Soil Type	Max T°	Min T°	pH
Adiyo	2573	2042.43	Clay loam	23.11	14.07	5.2
Doyogena	2535	1823.13	Clay loam	24.49	13.98	6.5
Hulla	2959	1255.39	Clay silt	25.15	13.99	5.0
Chencha	2985	1857.95	Nitosols	26.28	15.57	4.5

Source: climate data were taken from National Meteorology Agency (NMA).

**Table 5:** Description of oat genotypes used for the study.

Genotype Name	Genotype Code	Status
ILRI_5431A	G1	Promising
ILRI_5444A	G2	Promising
ILRI_5490A	G3	Promising
ILRI_5499A	G4	Promising
ILRI_5526A	G5	Promising
ILRI_5527A	G6	Promising
ILRI_15152A	G7	Promising
ILRI_15153A	G8	Promising
ILRI_16101A	G9	Promising
SRCPX80AB2291	G10	Released
SRCPX80AB2806	G11	Released

## Data Availability

The datasets collected for this current study are available from the corresponding author on reasonable request.
